# Sex Differences in Factors Affecting Depressive Symptoms in Older People in the Prefrailty Phase

**DOI:** 10.3390/ijerph17124207

**Published:** 2020-06-12

**Authors:** Eun Ju Lim

**Affiliations:** Red Cross College of Nursing, Chung-Ang University, Seoul 06974, Korea; dew7593@cau.ac.kr; Tel.: +82-2-820-5996

**Keywords:** depressive symptom, frailty, sex, older

## Abstract

Depressive symptoms experienced late in life have considerable effects on the prevalence of comorbidity with physical and cognitive disabilities. By identifying and intervening on modifiable indicators for depression in prefrail older individuals, progression to the frailty phase can be delayed, and physical, psychosocial, and cognitive health problems of older people can be reduced and prevented. This study assessed sex differences in factors affecting depressive symptoms in older people in the prefrailty phase in Korea. Data from the 2014 National Survey of Older Koreans revealed 1706 women and 662 men in the early stages of old age. Regression analysis showed that economic status, number of medications, social support, nutritional status, and fear of falling collectively accounted for 39.0% of the variance among older men. Using the same analysis method, economic status, number of chronic diseases, number of medications, chewing discomfort, regular exercise, social support, mobility, nutritional status, and fear of falling collectively accounted for 37.5% of the variance among older women. Based on differences in characteristics with respect to the sex of older people in the prefrailty phase, public health workers in the community should consider sex differences when planning a frailty management program.

## 1. Introduction

With a progressively aging population worldwide, there is an increased interest in healthy aging and a growing focus on the concept of frailty. Frailty refers to a state wherein one cannot properly respond to stress owing to aging and chronic diseases [1,2]. In a systematic review [2], the prevalence of frailty was approximately 9.9% in the population aged ≥65 years, and 44.2% of individuals were found to be in the prefrailty phase. The physical frailty phenotype, a measure for physical frailty first described by Fried et al., was developed based on cardiovascular health study indicators with a view to understanding frailty only from physical perspectives [1]. Frailty was defined as a clinical syndrome in which three or more of the following criteria were met: unintentional weight loss (10 lbs in the past year), self-reported exhaustion, resistance, slow walking speed, and low physical activity [1]. The prefrailty phase is the stage before the full manifestation of frailty [3], and this phase can be regarded as an important period that determines successful aging. Sezgin et al. [3] proposed a comprehensive definition suggesting that prefrailty is a multidimensional concept, an early and reversible risk state before frailty that can lead to negative healthcare outcomes, which is defined operationally by existing frailty screening and assessment tools. For older people who have entered the frailty phase, it is possible to slow down the progression of the condition; however, it is difficult to revert it back to the prefrailty phase [3]. Although diet or exercise therapy can be applied to older people in a state of physical frailty, its effects are limited. Because frailty is caused by the functional decline of multiple organs, it is difficult to improve it using a single treatment method [4]. For older people in the prefrailty phase, proper management can prevent progression into the frailty phase, and such older individuals tend to have a high post-treatment recovery rate [5]. Therefore, multifaceted research is needed on frailty-related physical, psychosocial, and cognitive characteristics of older people at a risk of entering the frailty phase to help those in the prefrailty phase to maintain or recover their current health and help them continue an independent life.

Depressive symptoms are a significant variable in predicting frailty and prefrailty, with higher levels of depressive symptoms leading to an increased chance of progressing from the prefrailty phase to the frailty phase [6], and older people diagnosed with depression are significantly more likely to become frail than those without depression [7,8]. As frailty progresses, maintaining homeostasis becomes impossible because of sudden decline in the physiological reserves of multiple organs [4] and increased levels of depression lead to decreased mobility and increased risks of falls, injuries, physical disabilities, hospitalization, and death [1]. However, the prevention of frailty can delay death in older people by 3–5% [9], implying the possibility to prevent diseases and death by maintaining or improving one’s function through appropriate interventions.

Depressive symptoms for the community-dwelling older people are related to physical, psychological, and cognitive functions such as frailty [10], physical function [11], nutritional status [12], fear of falling [13], and cognitive function [14] and sex differences between older men and women [11,14,15]. Throughout their life, women spend a lot of time parenting and doing domestic chores, and such social expectations lead women to experience inequalities in opportunities and resources [16]. Even in old age, women experience more social exclusion, such as economic poverty and lack of educational opportunities, than men, resulting in higher levels of depression [14]. The difference in the level of depression according to sex is expected to show in prefrail older people who experience difficulties in daily living and participating in social activities because of their lower level of physiological function; thus, it is necessary to consider sex when examining the factors affecting depression. Applying the concept of function based on clinical geriatrics, functional decline owing to frailty refers to declines in physical, cognitive, and psychosocial functions, which lead to an irreversible increase in health risks [17]. However, the prefrailty phase is a reversible state of health, and appropriate interventions can prevent diseases and death through the maintenance and improvement of bodily functions. While frailty is caused by a complex interaction between declining physical and psychosocial functions, only its physical aspect has been emphasized until now, with little interest in its psychosocial aspect. As another notable point, Crispell and Frey categorized older people aged 65–74 years into the young-old group and those aged ≥75 years into the old-old group, compared their characteristics, and reported differences in health status and social relationships according to age groups even within the older people group [18]. All aspects of health-related quality of life in old age are based on the level of healthcare provided in the early stages of old age. Therefore, this study was conducted for men and women in the early stages of old age to draw attention to the necessity of healthcare from the early stages of old age for a generally healthy life in old age.

Early screening of older people in the prefrailty phase and identification of characteristics defining such individuals are important in the development of clinical guidelines and nursing interventions that minimize the risk of progression into the frailty phase and development of clinical complications and disabilities. Thus, to delay or prevent the progression from the prefrailty phase to the frailty phase, this study aimed to propose a response strategy for frailty prevention by investigating depression-related sex differences among the prefrail older people and the basis for a preventive approach toward depression.

## 2. Materials and Methods

### 2.1. Ethical Approval and Consent

This study assessed data from the Korean Senior Citizens Survey executed by the Korea Institute for Health and Social Affairs (KIHASA) after submitting research proposals for their use. The study was approved by the Institutional Review Board of the C University to which the author was affiliated (Number: 1041078-201707-HR-136-012019-001). The author conformed to all ethical guidelines regarding plagiarism, misconduct, data fabrication and/or falsification, double publication and/or submission, and redundancy.

### 2.2. Design and Sample

This study used the data from the KIHASA that is organized by the Ministry of Health and Welfare in 2014. To be representative of the Korean population, the KIHASA focuses on Koreans aged ≥65 years from households selected by stratified two-stage cluster sampling based on the geographical area. The primary sampling unit is 90% of the sample enumeration district based on the 2010 Population and Housing Census data, the secondary sampling unit is composed of households in the enumeration district, and the final survey unit is composed of all people aged ≥65 years in the households. A questionnaire survey was conducted, and the survey used cross-sectional research data. The researcher submitted a data request letter to use the original data, research plan, and written pledge and obtained approval for data use from the KIHASA.

Data were stratified according to seven metropolitan cities and nine provinces. The nine provinces were divided into 25 classes with stratification into dongs (neighborhoods) and eups/myeons (towns/townships) for sample collection. The participants for this study comprised 1706 women and 662 older men who were in the prefrailty phase and who were selected from among 10,451 participants after excluding older people aged ≥75 years, who were frail and nonfrail, and who submitted missing data. The number of subjects was deemed sufficient for this research as the minimum number required was 92 [19] based on an F-test conducted with values calculated with G*Power 3, which yielded an effect size of 0.15 (medium), significance of 0.05, and power of 0.80.

### 2.3. Measures

#### 2.3.1. Frailty

This study used the Korean version of the FRAIL (K-FRAIL) scale, developed by Jung et al. [20] based on the study of Fried et al. [1]; (1) Fatigue: Did you feel tired in the past month? 1 = always, 2 = often, 3 = sometimes, 4 = seldom, or 5 = not at all (1 point for answering 1 or 2, 0 points otherwise); (2) Resistance: Is it difficult to walk 10 steps up a stair without help or rest? Yes = 1 point or No = 0 point; (3) Ambulation: Is it difficult to walk 300 m without help or rest? Yes = 1 point or No = 0 point; (4) Illness: Have you been diagnosed with a chronic illness (high blood pressure, diabetes, cancer, chronic lung disease, myocardial infarction, cardiac insufficiency, angina, asthma, arthritis, cerebral infarction, kidney disease) by a doctor? 0 points for 0–4 illnesses or 1 point for 5–11 illnesses; and (5) Loss of weight: 1 point for a weight loss of ≥5% over the past year or 0 points for <5% weight loss. According to the K-FRAIL scale, individuals with ≥3 points are deemed frail, 1–2 points are prefrail, and 0 points are normal. The sensitivity of the K-FRAIL scale was 0.90.

#### 2.3.2. Mobility

Mobility was measured using the tools described by Lee et al. [21]. The scale comprised five questions, with two regarding upper limb mobility and three regarding lower limb mobility. For the upper limb mobility, the flexibility and muscular power of shoulder joints were measured, and for the lower limb mobility, the endurance, balance, walking, and flexibility were measured. Here, the participant reaches up over his/her head and lifts or carries a bag of rice (8 kg), walks 400 m, and climbs 10 steps of stairs without stopping, stooping, crouching, or kneeling. The items were scored from 0 to 3 corresponding to the degree of difficulty in performing the individual functions (0, full help needed; 1, a lot of difficulty; 2, some difficulty; 3, no difficulty). The total score was divided by the number of questions and by the highest score, namely, 3 points. Then, the partial score was multiplied by 40 for the upper limb score and by 60 for the lower limb score to obtain a total score of 100 points. Higher points indicated better mobility. The measures have been tested for validity and reliability in a community-dwelling older population [22]. Cronbach’s ⍺ value during the development of the mobility scale was 0.87 and that for this study was 0.74.

#### 2.3.3. Nutritional Status

This study used the Korean version of the Nutritional Screening Initiative (NSI) checklist, which has been translated and modified [23] to adapt to the Korean context, to examine the nutritional status of older people. The NSI checklist was developed in collaboration with 30 relevant associations, including the American Academy of Family Physicians, American Dietetic Association, and National Council on Aging in the United States. The NSI checklist includes 10 differently weighted yes/no items associated with the nutritional wellbeing of an older individual. The cumulative scores range from 0 to 21. In this study, individuals with total scores between 0 and 2 belonged to the well-nourished group and those with scores ≥ 6 were classified into the malnourished group. Higher scores indicated worse nutrition status. Cronbach’s α-value for this measure was 0.75 in a previous study [23] and 0.72 in the present study.

#### 2.3.4. Depressive Symptoms

The depression measurement tool used was the Short Geriatric Depression Scale that was developed by Yesavage et al. [24]. The tool was modified by Cho et al. [25] and accepted because of its reliability and feasibility in Korea. For each question, “Yes” was 1 point and “No” was 0 points. The five positive questions were converted into scores after inverse coding and summed up. The score ranged from 0 to 15 points. Higher points indicated higher depression levels. The reliability Cronbach’s ⍺ value was 0.84 at the time of development and 0.91 for this study.

#### 2.3.5. Fear of Falling

Fear of falling was rated and assigned 1 point for a response of “I don’t worry at all” and 3 points for “I worry very much.” A higher score indicated a greater fear of falling. Although the inherent weakness owing to single-item measures was recognized, the single-item measurement method was simple and suitable for older people who may have decreased cognitive functions and have a high correlation with the efficacy of fall prevention [26].

#### 2.3.6. Cognitive Function

To evaluate cognitive function, I used the Mini-Mental Status Examination from the Korean version of the CERED assessment packet (MMSE-KC) [27]. Items in this examination addressed orientation to time and place (10 points), immediate recall (3 points), attention (5 points), delayed recall (3 points), language ability (6 points), configuration capability (1 point), and comprehension and judgment (2 points). Total scores ranged from 0 to 30 points, with higher scores indicating higher cognition levels. Summary scores were evaluated relative to norms for sex, educational level, and age. The MMSE-KC was standardized, and the κ coefficient, indicating agreement between the psychiatrist’s diagnosis and the MMSE-KC score, was 0.63 [27]. In this study, Cronbach’s α value was 0.80.

#### 2.3.7. Other Variables

Data on the following sociodemographic characteristics were collected during the interview by using a structured questionnaire: economic status (lower, middle, upper), number of chronic diseases diagnosed by a physician (<3, ≥3), number of medications over 3 months (<3, 3–8, ≥9), chewing discomfort (none, slightly uncomfortable, very uncomfortable), regular exercise (yes, no), and self-reported social support (not satisfied, a little satisfied, very satisfied).

### 2.4. Statistical Analysis

Data were analyzed using SPSS for Windows (version 18.0; SPSS, Chicago, IL, USA). To reduce selection bias and ensure representativeness, weighted values were calculated using composition ratios by sex and age. The t-test and χ^2^-test were conducted to verify the homogeneity of the participants’ sociodemographic characteristics. Independent sample t-tests were performed to determine the average difference between measurement variables (mobility, nutritional status, depressive symptoms, fear of falling, cognitive function) by separating variables by sex. After analyzing the correlation between depression and study variables, multiple regression analysis was performed using the variables with significant relationships as independent variables.

## 3. Results

### 3.1. Sociodemographic Characteristics

**Figure 1:**
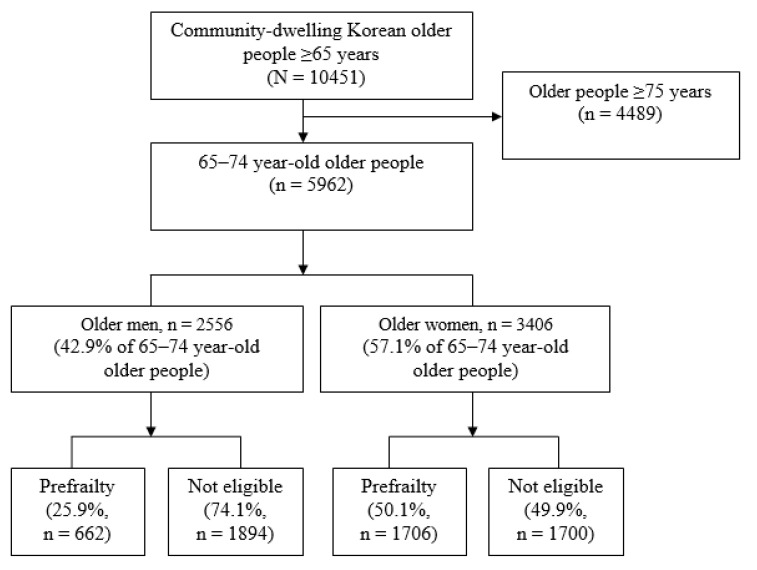
Flow diagram.

Among the prefrailty phase subjects, 25.9% were older men and 50.1% were older women (Figure 1). The average age of 2368 subjects, which consisted of 662 men and 1706 women (Table 1), was 69.2 (±8.69) years, and 1261 (53.3%) were older people whose economic level was “low.” In total, 1457 (61.5%) subjects had “≥3” chronic diseases, 1000 (42.2%) consumed “3–8 drugs” for >3 months, and 1027 (43.4%) answered “slightly uncomfortable” chewing function. Moreover, 1194 (50.4%) subjects participated in regular exercise, and 1257 subjects were (53.1%) “very satisfied” with the self-reported social support level. In this study, homogeneity was maintained because no difference was found between sexes according to the demographic characteristics of the older men and women.

### 3.2. Sex Differences in Mean Scores of Study Variables

Table 2 shows the mean score of each variable with respect to sex. Mobility (t = 3.105, *p* = 0.002), fear of falling (t = −10.602, *p* < 0.001), and cognitive function (t = 7.568, *p* < 0.001) showed significant differences between the two groups. In contrast, nutritional status (t = 0.219, *p* = 0.827) and depressive symptoms (t = −1.803, *p* = 0.072) were not significantly different between the two groups.

### 3.3. Sex Differences in the Correlation between Study Variable and Depressive Symptoms

Table 3 shows the results after analyzing the correlation between depression and research variables in older men and women. In men, depression, mobility (r = −0.318, *p* < 0.001), and cognitive function (r = −0.174, *p* < 0.001) were statistically significantly inversely correlated, and depression, nutritional status (r = 0.391, *p* < 0.001), and fear of falling (r = 0.404, *p* < 0.001) were found to have a statistically significant correlation. In older women, depression, mobility (r = −0.341, *p* < 0.001), and cognitive function (r = −0.109, *p* < 0.001) were statistically significantly inversely correlated, and depression, nutritional status (r = 0.398, *p* < 0.001), and fear of falling (r = 0.309, *p* < 0.001) were found to have a statistically significant correlation.

### 3.4. Sex Differences in Factors Influencing Depressive Symptoms

Table 4 shows the sex differences in factors affecting depression. For the group comprising older men, economic status (β = −0.142, t = −4.346, *p* < 0.001), number of medications (β = 0.131, t = 3.725, *p* < 0.001), self-reported social support (β = −0.242, t = −7.243, *p* < 0.001), nutritional status (β = 0.180, t = 5.167, *p* < 0.001), and fear of falling (β = 0.173, t = 4.951, *p* < 0.001) collectively accounted for 39.0% of the variance of depression (F = 36.170, *p* < 0.001). For the group comprising older women, economic status (β = −0.193, t = −9.513, *p* < 0.001), number of chronic diseases (β = 0.104, t = 4.678, *p* < 0.001), number of medications (β = 0.057, t = 2.547, *p* = 0.011), discomfort in chewing (β = 0.056, t = 2.646, *p* = 0.008), regular exercise (β = 0.043, t = 2.176, *p* = 0.030), self-reported social support (β = −0.208, t = −10.473, *p* < 0.001), mobility (β = −0.091, t = −3.662, *p* < 0.001), nutritional status (β = 0.169, t = 7.513, *p* < 0.001), and fear of falling (β = 0.125, t = 6.089, *p* < 0.001) collectively accounted for 37.5% of the variance of depression (F = 85.896, *p* <0.001).

## 4. Discussion

Depressive symptoms experienced late in life have expansive effects on the prevalence of comorbidity with accompanying physical and cognitive disabilities. By identifying and intervening on modifiable indicators for depression in prefrail older people, progression into the frailty phase can be delayed, and the physical, psychosocial, and cognitive health problems of older people can be reduced and prevented. The significance of this study was its identification of sex-related differences in factors affecting depression in prefrail older people.

The results indicated that among older people in the prefrailty phase, 25.9% were men and 50.1% were women, which confirms the results of Raji et al. [28] who found more older women in the prefrailty stage than men. In contrast, 39.7% of the community-dwelling older people were found to be prefrail; this result is different from that of the study conducted to assess the effectiveness of a frailty prevention intervention program for older people aged ≥70 years [29], that is, 49% of older people were in the prefrail stage. Both values fall within the 35%–50% range of prefrailty prevalence among older people aged ≥65 years, as reported by Fernández-Garrido et al. [30]. The discrepancy between these distributions may be attributed to the differences in the average age, race, and culture of the research subjects. Care of individuals in the prefrailty stage can prevent progression into frailty through a healthy lifestyle, resulting in a high potential for recovery of their health [4]; thus, early screening and management of prefrail older people are critical.

Statistically significant differences were observed in the average scores for mobility, fear of falling, and cognitive function among variables measured for prefrail older men and women. According to Lim [11], upper and lower limb mobility for community-dwelling older men were statistically significantly higher than those of older women, as confirmed in the present study. The average score for fear of falling among the prefrail older people examined in this study was above medium, signifying decreased or decreasing physical functions such as static balance and complex movements [15]. Since older people are more prone to frailty owing to lowered physical function, those at the prefrailty phase must be categorized and managed as a high-risk group that requires preventive intervention for falling. While no significant difference was found between men and women with respect to depressive symptoms, the latter showed a higher level of depression. These results are higher than those reported by Choi et al. (2011) [15], where the level of depression was measured with the same scale among frail older people aged ≥65 years. Since older women are reported to be more likely to have depression owing to their disadvantaged living conditions in terms of economic, social, and health aspects than older men [16], sex should be considered when administering therapeutic interventions for depression.

Depressive symptoms were inversely related to mobility and cognitive function and positively correlated to nutritional status and fear of falling in both older men and women in the prefrailty phase. This indicated that increased depressive symptoms result in lower physical and cognitive functions, worse nutritional status, and increased fear of falling. Such results confirm the findings of previous studies that found depressive symptoms to be inversely related to upper and lower limb mobility [11] and cognitive function [14].

Common factors affecting depressive symptoms of both prefrail men and women were economic level, number of medications administered for ≥3 months, social support, nutritional status, and fear of falling, among which social support had the greatest effect on depression. According to a study by Kim and Choi [31], which included 492 subjects aged ≥65 years, the examined factors affecting depression among the urban older women included being economically inactive, having chronic illnesses, and having low social support, and this had a greater effect on depression in a similar context to the results of the present study. Fear of falling at baseline statistically significantly predicted depression at 12-month follow-up assessment after adjusting for age, sex, marital status, education, and depressive symptom at baseline [13]. Moreover, social functioning mediated the impact of fear of falling on depressive symptoms [13]. In contrast, a study by Makizako et al. [32] found that cognitive function did not affect depression. According to a longitudinal study [33], when various physical disabilities and functions were tracked based on measurements, the beginning or worsening of depression was found to be positively related to progression into frailty. Furthermore, a longitudinal cohort study that examined 3025 community-dwelling older people without depression over 15 months [32] revealed that depressive symptoms were observed in 7.5% of the subjects, with generally low self-assessment of health and frailty being independent predictors. Such results indicate a reciprocal relationship between depressive symptoms and frailty for older people [10]. Thus, the paradigm shift of conceptualizing and measuring depressive symptoms and frailty will affect not only the approach to prevention and intervention but also its pleiotropic effect.

Among factors affecting the depressive symptoms of prefrail older people, differences were found in the number of chronic diseases, chewing discomfort, regular exercise, and mobility between the sexes. Depressive symptoms intensified as the number of chronic diseases increased in older women, but this association was not found in older men [34]. Older people with chewing discomfort were 1.83 times more likely to have depressive symptoms than healthy older people [35], showing a pattern similar to the findings of the present study. It is thought that the higher the number of chronic diseases and the higher the level of chewing discomfort, the lower is the perceived health level, resulting in depression. In this regard, oral care projects should be carried out regularly so that older women can maintain healthy teeth and oral conditions, and preventive management of chronic diseases is necessary. Older women are more susceptible to sarcopenia or osteoporosis than older men, which increase the likelihood of experiencing changes in body function. In older people, a decrease in mobility due to a decline in physical ability has been reported to increase the level of depression because of social isolation and lowered life satisfaction [32], which is consistent with the results of this study. Depressive symptoms are thought to be aggravated, while the decrease in mobility directly caused limitations in activities of daily living. A study reported that regular exercise is effective in alleviating depressive symptoms in community-dwelling older people and inpatients [36], supporting the findings of this study. Customized exercise programs to improve physical fitness and physical functions should be developed and applied for prefrail older women [37], as they have been identified to improve mobility and reduce the fear of falls in older women who have received muscle strength training and balance training.

Despite its valuable findings, the study has some limitations that need to be addressed. First, the results may not be generalizable to all older people, including those aged ≥75 years, because the subjects were limited to those who met the criterion for older people in the early stages of old age. Second, owing to the cross-sectional design, the causal inferences among the variables cannot be proven; thus, the findings of this study need to be interpreted with caution. However, the apparent sex differences in the predictors of depressive symptoms among older people indicate that prevention programs will need to reflect these differences through tailored assessments and customized interventions.

### Implications

The results of this study are expected to influence both research and practice on the management of depression and prevention of frailty in prefrail people in the early stages of old age. In terms of managing depression, in addition to factors that commonly affect depressive symptoms, the number of chronic diseases, chewing discomfort, regular exercise, and mobility were significant factors only in older women, confirming that in reducing depressive symptoms, health intervention considering sex differences is necessary. Social support to avoid the exacerbation of depressive symptoms can serve as a buffer for the psychological state of the subjects, providing significant physical and psychosocial benefits to older people. The findings of this study are also important in identifying the depression status of prefrail people in the early stages of old age and in raising the awareness of stakeholders on the role of social support in reducing symptoms.

In this study, 25.9% of older men and 50.1% of older women were found to be in the prefrailty phase; thus, there were more women in the potential frailty phase than men. As the number of older people in the frailty phase increases with age [4], when the entire older population was considered, the distribution level of older women is expected to further increase. With regard to frailty prevention, to ensure that vulnerable older people in the prefrailty phase receive help in multifaceted aspects of demography, physical health, social resources, and mental health, local health professionals should focus on the development of intervention programs reflecting these needs.

## 5. Conclusions

Depressive symptoms are community mental health problems that must be addressed, as they have a considerable effect on the overall health and quality of life of the older population. In this study, the number of chronic diseases, chewing discomfort, regular exercise, and mobility were the factors that affect depressive symptoms of people in the early stages of old age, which were significant only among women. As social support was found to be the primary factor affecting depressive symptoms in both older women and men, public health care workers must introduce community resources and promote program participation if older people have difficulties in maintaining a social relationship with their families. Although interventions to prevent frailty in older people and promote healthy aging have focused on regular exercise, balanced diet, and cognitive training up until now, lifestyle interventions in such areas should additionally consider psychosocial aspects in the future. Moreover, well-grounded frailty prevention programs must incorporate the results of studies that examine frailty and depression from a broader perspective.

## Figures and Tables

**Table 1:** Sociodemographic characteristics (n = 2368).

Variable	Total (n = 2368) N (Weighted %)	Male (n = 662) N (Weighted %)	Female (n = 1706) N (Weighted %)	t/χ^2^ (*p*)
age (years) mean ± standard deviation	69.2 ± 8.69	69.3 ± 5.99	69.1 ± 9.53	0.736 (0.462)
economic status				2.032 (0.362)
lower	1261 (53.3)	337 (50.9)	924 (54.2)	
middle	1036 (43.8)	304 (45.9)	732 (42.9)	
upper	71 (3.0)	21 (3.2)	50 (2.9)	
number of chronic diseases				17.399 (0.415)
<3	911 (38.5)	299 (45.2)	612 (35.9)	
≥3	1457 (61.5)	363 (54.8)	1094 (64.1)	
number of medications (>3 months)				0.340 (0.844)
<3	628 (26.5)	170 (25.7)	458 (26.8)	
3–8	1000 (42.2)	282 (42.6)	718 (42.1)	
≥9	740 (31.3)	210 (31.7)	530 (31.1)	
discomfort in chewing				3.503 (0.174)
none	935 (39.5)	246 (37.2)	689 (40.4)	
slightly uncomfortable	1027 (43.4)	289 (43.7)	738 (43.3)	
very uncomfortable	406 (17.1)	127 (19.2)	279 (16.4)	
regular exercise				0.805 (0.384)
yes	1174 (49.6)	338 (51.1)	836 (49.0)	
no	1194 (50.4)	324 (48.9)	870 (51.0)	
self-reported social support				17.577 (0.162)
not satisfied	239 (10.1)	92 (13.9)	147 (8.6)	
a little satisfied	871 (36.8)	250 (37.8)	621 (36.4)	
very satisfied	1257 (53.1)	319 (48.3)	938 (55.0)	

**Table 2:** Sex differences in mean scores of study variables of older people in the prefrailty phase (n = 2368).

Variables	Male (n = 662) Mean ± SD	Female (n = 1706) Mean ± SD	t (*p*)
mobility (range: 0–100)	74.08 ± 21.07	69.63 ± 20.22	3.105 (0.002)	
nutritional status (range: 0–21)	3.68 ± 2.87	3.65 ± 2.89	0.219 (0.827)
depressive symptoms (range: 0–15)	5.72 ± 4.79	6.11 ± 4.70	−1.803 (0.072)	
fear of falling (range: 1–3)	2.17 ± 0.73	2.50 ± 0.60	−10.602 (<0.001)
cognitive function (range: 0–30)	24.74 ± 4.88	22.99 ± 5.42	7.568 (<0.001)

**Table 3:** Sex differences in the correlation between study variable and depressive symptoms of older people in the prefrailty phase (n = 2368).

Variables	Depressive Symptoms	
	Male (n = 662) r (*p*)	Female (n = 1706) r (*p*)
mobility	−0.318 (<0.001)	−0.341 (<0.001)
nutritional status	0.391 (<0.001)	0.398 (<0.001)
fear of falling cognitive function	0.404 (<0.001) −0.174 (<0.001)	0.309 (<0.001) −0.109 (<0.001)

**Table 4:** Sex differences in factors affecting depressive symptoms of older people in the prefrailty phase (n = 2368).

Variables	Depressive Symptoms			
	Male (n = 662)		Female (n = 1706)	
	β	t (*p*)	β	t (*p*)
age	−0.026	−0.838 (0.403)	−0.015	−0.796 (0.426)
economic status	−0.142	−4.346 (<0.001)	−0.193	−9.513 (<0.001)
number of chronic diseases	0.055	1.556 (0.120)	0.104	4.678 (<0.001)
number of medications	0.131	3.725 (<0.001)	0.057	2.547 (0.011)
discomfort in chewing	0.059	1.784 (0.075)	0.056	2.646 (0.008)
regular exercise	0.053	1.666 (0.096)	0.043	2.176 (0.030)
self-reported social support	−0.242	−7.243 (<0.001)	−0.208	−10.473 (<0.001)
mobility	−0.072	−1.730 (0.084)	−0.091	−3.662 (<0.001)
nutritional status	0.180	5.167 (<0.001)	0.169	7.513 (<0.001)
fear of falling	0.173	4.951 (<0.001)	0.125	6.089 (<0.001)
cognitive function	−0.058	−1.850 (0.065)	−0.028	−1.424 (0.154)
R^2^	0.390		0.375	
F (*p*)	36.170 (<0.001)		85.896 (<0.001)	
